# Sepsis-Induced Immunosuppression in Neonates

**DOI:** 10.3389/fped.2018.00357

**Published:** 2018-11-29

**Authors:** Julie E. Hibbert, Andrew Currie, Tobias Strunk

**Affiliations:** ^1^Centre for Neonatal Research and Education, University of Western Australia, Perth, WA, Australia; ^2^School of Veterinary and Life Sciences, Murdoch University, Perth, WA, Australia; ^3^Neonatal Directorate, King Edward Memorial Hospital for Women, Subiaco, WA, Australia

**Keywords:** neonates, preterm infant, innate immunity, adaptive immunity, immune cell function, sepsis, infection, immunosuppression

## Abstract

Neonates, especially those born preterm, are at increased risk of sepsis and adverse long-term effects associated with infection-related inflammation. Distinct neonatal immune responses and dysregulated inflammation are central to this unique susceptibility. The traditional separation of sepsis into an initial hyper-inflammatory response followed by hypo-inflammation is continually under review with new developments in this area of research. There is evidence to support the association of mortality in the early acute phase of sepsis with an overwhelming hyper-inflammatory immune response. Emerging evidence from adults suggests that hypo- and hyper-inflammation can occur during any phase of sepsis and that sepsis-immunosuppression is associated with increased mortality, morbidity, and risk to subsequent infection. In adults, sepsis-induced immunosuppression (SII) is characterised by alterations of innate and adaptive immune responses, including, but not limited to, a prominent bias toward anti-inflammatory cytokine secretion, diminished antigen presentation to T cells, and reduced activation and proliferation of T cells. It is unclear if sepsis-immunosuppression also plays a role in the adverse outcomes associated with neonatal sepsis. This review will focus on exploring if key characteristics associated with SII in adults are observed in neonates with sepsis.

## Inflammation and sepsis—a new paradigm?

Sepsis, defined as life-threatening organ dysfunction caused by a dysregulated host response to infection, represents an enormous burden affecting more than 30 million people with potentially 6 million associated deaths per year (1). Until recently, adult sepsis complicated by organ dysfunction, was termed severe sepsis, but is now represented by an increase in the Sequential Organ Failure Assessment (SOFA) score, secondary to the infection cause (2). The SOFA score, based on respiratory, cardiovascular, hepatic, coagulation, renal, and neurological systems, determines the extent of organ function and an increase of two points or more is associated with in-hospital mortality of >10% (2). Septic shock is defined as sepsis with circulatory and cellular/metabolic abnormalities that substantially increase mortality (2). Recent evidence from critically ill adults with sepsis and septic shock suggests the extent of recovery from sepsis may depend on the host's ability to orchestrate both the pro-inflammatory and hypo-inflammatory responses to achieve immunological homoeostasis following infection (3, 4). Hotchkiss et al., described three potential inflammatory responses to sepsis, and acknowledged that the immune response to sepsis is determined by many factors, including pathogen virulence and comorbidities (4). Firstly, at the onset of sepsis the pro-inflammatory response can dominate, even though both the pro-inflammatory and anti-inflammatory responses are initiated, and lead to an overwhelming hyper-inflammatory state that may cause multiple organ dysfunction and death within 1–2 days. Secondly, in patients with impaired immune responses due to comorbidities, the hyper-inflammatory phase may be absent or reduced and a profound anti-inflammatory state may occur, which may lead to further impaired immunity with increased risk of nosocomial infections and higher risk of death 10–14 days following sepsis onset. Thirdly, the immune response cycles between hyper-inflammatory and hypo-inflammatory states and death can occur in either state. With this response, there is an increased probability of the patient developing overwhelming immunosuppression as the infection persists (4).

There is increasing evidence to support the role of immunosuppression in sepsis (4). Critically ill adults with sepsis and septic shock may develop *sepsis-induced immunosuppression (SII)*, a phenomenon of persistent systemic hypo-inflammation that compromises many immune functions, prevents bacterial elimination and immune homeostasis (3–7). Importantly, SII is associated with increased risk of multi-organ failure and mortality, and ongoing immunosuppression results in prolonged (10–14 days) susceptibility to secondary viral and bacterial infections (3–6, 8).

Immunologically, SII in adults is incompletely characterised, but is commonly associated with altered functions of the complex network of innate and adaptive immune responses to infection. Specifically, neutrophils, monocytes, macrophages, and dendritic cells (DCs) display a prominent shift of phenotype and function toward an impaired inflammatory response. This further includes decreased bactericidal defences in neutrophils (including oxidative burst and low intracellular expression of myeloperoxidase and lactoferrin), reduced neutrophil chemotaxis, biased anti-inflammatory cytokine secretion with an increased interleukin (IL)-10 to tumour necrosis factor alpha (TNFα) ratio. There is also reduced expression of the major histocompatibility complex (MHC) II cell surface receptor human leukocyte antigen-DR (HLA-DR) and consequently impaired antigen-presenting capacity of DCs and monocytes (3, 4, 6, 7). Changes to the adaptive immune response in adults with SII include: reduced activation of T cells through diminished cell surface expression of the co-stimulatory molecules CD80 and CD86 on antigen presenting cells (APCs); inhibition of T cell proliferation due to expansion of cell populations with immunosuppressive function, such as immature neutrophils, myeloid-derived suppressor cells (MDSCs), and regulatory T cells (Tregs); reduced effector functions of T cells, B cells, and natural killer cells; and T cell exhaustion, typified by decreased T cell activation, reduced ability to produce cytokines, and decreased cytotoxic functions (3, 4, 6, 7). Sepsis-induced apoptosis of DCs, CD4+ and CD8+ T cells and B cells occurs in primary immune organs such as blood, bone marrow, spleen, and thymus, resulting in an overwhelming depletion of immune cells (3, 4, 6, 7).

The suppressive effect of endotoxin tolerance, induced by repeat or long-term exposure to bacterial endotoxins, like lipopolysaccharide (LPS), mediates immune dysfunction through reprogramming of cell signalling and is associated with immunosuppression observed in the later-stage of adult sepsis (7, 9). Leukocytes from adult patients with sepsis behave similarly to *in vitro* endotoxin-tolerised cells, with a reduced responsiveness to produce cytokines, especially TNFα, upon re-challenge with LPS (10). The molecular mechanism is unclear, but Pena and colleagues have recently identified an endotoxin tolerance gene signature that may predict sepsis and organ dysfunction in adults with sepsis (9). Murine macrophages challenged with Gram-positive bacteria can also induce endotoxin tolerance, termed as cross-tolerance, to a lesser extent than LPS (11), but no associated gene signature has yet been reported.

## Sepsis-induced immunosuppression and the neonate

Neonatal immune development is complex, incompletely understood and orchestrated by many factors, including intra- and extra-uterine exposure to antigens and commensal organisms (12–16). Immune development in infants born preterm (<37 weeks' gestation) may be further altered by perinatal exposures to corticosteroids and antibiotics and the unique environmental influences associated with prolonged hospital stay (e.g., mechanical ventilation, use of indwelling plastic devices, parenteral nutrition, invasive procedures, and exposure to nosocomial microbes) (14, 15, 17–19).

Despite advances in neonatal care, sepsis remains a significant cause of morbidity among neonates and is one of the most common causes of neonatal death, accounting for over four-hundred and twenty thousand deaths per year (20). Sepsis is a common complication that affects up to 40% of neonates born <28 weeks' gestation (21, 22). Chronic long-term morbidities, such as lung disease and neurodevelopmental impairment, are further increased among infants who acquire nosocomial sepsis (23, 24). Inflammation-related brain injury and the associated long-term effects are clearly evident in preterm infants with sepsis (23), and have also been observed in adults with sepsis (25).

The increased risk of sepsis-associated morbidity and mortality in neonates is largely attributed to immature innate immune functions resulting in dysregulated pro-inflammatory responses to systemic infection—often referred to as a “cytokine storm” (26–30). The mortality rate in infants with sepsis is 10–16%, with 50–57% of neonates die within the first 3 days of sepsis onset, 12–20% within 4–7 days and 23–39% after 7 days (31, 32). Similar to adults (4), there is strong evidence to support the association of mortality in the acute phase, i.e., within first days of sepsis, with a dysregulated pro-inflammatory immune response (28–30, 33, 34). However, emerging evidence suggests that the immature immune response to infection in neonates is characteristically similar to the endotoxin tolerance phenotype observed in critically ill adults with sepsis (7, 35). This is evident despite Gram-positive bacteria being the major causative organisms in neonatal sepsis (36), unlike in adult sepsis (37). In the neonatal setting, the risk of sepsis may be mediated by a relative inability to initiate appropriate hyper-inflammatory responses which, along with a predominant hypo-inflammatory response, actively causes immunosuppression (35). In keeping with findings in adult SII, Gervassi and colleagues proposed that the distinct neonatal responses to invasive microbes, as well as to vaccines, may be at least partly explained by active immune suppression, such as inhibition of T cell proliferation and function by Tregs and MDSCs, and the potential for B cells to skew the immune response toward an anti-inflammatory T helper 2 response (38).

Sepsis-induced immunosuppression has not yet been defined in neonates. In adults, SII signifies sepsis severity, septic shock, and mortality (3, 4, 6, 7). Identifying immunosuppression in neonates may not be as straight forward for several reasons, namely: the lack of a globally accepted definition for neonatal sepsis, including grading of severity; the distinct patterns of immune development; and the sparse data available on immune function and response to infection at the time of neonatal sepsis.

Firstly, neonatal sepsis, especially in those born preterm, is not clearly defined. The recently updated Third International Consensus Definitions for sepsis and septic shock in adults are not applicable to children, infants and neonates (2, 39, 40), and there is no equivalent SOFA score for determining sepsis severity in neonatal sepsis. Further to this, the international paediatric consensus definition for sepsis specifically excludes preterm neonates (39) and performs poorly in term neonates (40). This has widespread implications not only for reporting incidence and prevalence of neonatal sepsis, but for clinical management (accurate diagnosis and appropriate treatment) and the short- and long-term impact on clinical outcomes (41, 42). Further to this, the lack of a clear neonatal sepsis definition creates a substantial barrier to identifying predictive markers for sepsis, and improving diagnostic accuracy and speed (41, 43). To date, there is no consensus definition of neonatal sepsis and the current “gold standard” of positive blood culture plus clinical symptoms for the definition of “confirmed sepsis” has significant limitations (41, 44, 45). To further complicate diagnosis, sepsis can also be classified as “clinical sepsis,” with a negative culture in a symptomatic newborn (41).

Secondly, development of immunoregulation is distinct in neonates compared with adults. This includes differences in: (a) absolute numbers of immune cells (e.g., lower neutrophil counts and higher natural killer cell counts in neonates) (12, 46, 47); (b) the proportions of immune cell subtypes (e.g., higher immature/total neutrophil ratio in neonates) (48, 49), and (c) levels of various immune plasma proteins (e.g., lower complement, immunoglobulin, antimicrobial peptide levels in neonates) (12, 13).

Lastly, there is limited data on neonatal innate immune responses during sepsis, and studies relating immune function to sepsis severity are lacking. Neonatal immune studies commonly utilise cord blood, which is not representative of the immune system at 1–3 weeks of age, when the most common form of neonatal invasive infection, late-onset sepsis (LOS), typically occurs (50). Data available from the time of sepsis can be limited by low number of neonates and confounded by multiple factors, such timing of sample collection, volume of blood sample collection, pathogenesis of the causative organism, time of sepsis onset [e.g., early-onset sepsis (EOS) <72 h after birth and LOS >72 h after birth], and sepsis definition (e.g., confirmed vs. clinical). Further to this, time from sepsis onset to death is poorly reported and therefore causative attribution not consistently possible.

Confirming and describing SII in neonates may be instrumental in better defining the immune pathophysiology of neonatal sepsis. This could also aid in the identification of unique biomarkers that could be of clinical utility for immunomonitoring, prediction of outcomes, or even targeted therapeutics. The remainder of this review focusses on characterising immunosuppression in neonatal sepsis and their associated clinical implications. The principal immune functions characterised include cytokine secretion, antigen presentation, expansion of immunosuppressive cells, effector cell function, and sepsis-induced immune cell apoptosis. Information on gestational age (GA), postnatal age at onset of sepsis, classification (i.e., confirmed and clinical sepsis) and age at sepsis-related death in many studies were incomplete and may confound the interpretation. Available data were included in this review and in Tables 1–5 and Supplementary Tables 1, 2. For clarity, when neonatal GA was not described in the publication, we considered any infant born ≥37 weeks' gestation as term and any infant born <37 weeks' gestation as preterm.

**Table 1:** Sepsis-induced immunosuppression—association of secreted cytokine concentration with sepsis severity in neonates and adults with sepsis.

**Adult or neonatal GA**	**Cohort sepsis characteristics (*n*) and mortality (if applicable)**	**Time of blood sampling and age at sepsis**	**Observation in septic cohort**	**References**
**TNFα**				
Adult	Organ dysfunction during sepsis: 24 - Organ failure recovery by day 4: 11 - Organ failure ongoing: 13	Blood samples were taken within 24 h of initial suspicion of sepsis and on hospital days 4 and 6Mean (median) age at sepsis 55 (55) years	Increased TNFα production capacity is associated with organ failure recovery	(51)
Adult	Septic shock: 38 - Survivors: 22 - Non-survivors: 16 Mortality within 28 days after diagnosis. Time from sepsis onset not described	Blood samples were taken on days 1–2, 3–4, 5–7, and 8–15 days following initial suspicion of sepsisMean age at sepsis 64 years (95% CI 59–69)	TNFα levels were increased in non-survivors compared to survivors, but not significantly	(52)
Term(GA range 37–42 weeks)	Clinical (*n* = 10) and confirmed (*n* = 3) LOS: 13 - Sepsis: 4 - Severe sepsis: 6 - Septic shock: 3	Blood sample was taken at initial suspicion of sepsisMedian age at sepsis: 10 days (IQR 7–22 days)	TNFα levels were not associated with sepsis severity	(53)
Mix of preterm andTerm(mean GA not described)	Sepsis: 50 (EOS: 41 and LOS: 9) - Survivors: 33 - Non-survivors:17 Non-sepsis inflammation: 50 Controls: 50 Time from sepsis onset to death not described	Blood samples were taken at sepsis evaluation (time 0) and on days 1 and 2 Age at sepsis not described	TNFα was significantly elevated in non-survivors, compared to survivors, at time 0, but not on days 1 or 2	(54)
Mix of preterm andTerm(mean GA 35.8 ± 4.1)	Confirmed sepsis: 26 (EOS *n* = 3 and LOS *n* = 13) - Survivors: 17 - Non-survivors: 9 Controls: 29 Mortality: EOS deaths <2 days: 5 LOS deaths >7 days: 4 Time from sepsis onset to death not described	Blood samples were taken at sepsis evaluation before antimicrobial therapy (time 0) and on days 3 and 7 Mean (±SD) age at sepsis:EOS 1.9 (±1.1) daysLOS 20.6 (±8.4) days	TNFα significantly increased progressively during sepsis in the non-survivors TNFα significantly decreased progressively during sepsis in the survivors	(55)
**IL-6**				
Adult	Septic shock: 20 SIRS: 11 Healthy controls: 10	Blood sample was taken within 24 h initial suspicion of sepsisAge at septic shock: 68 years	IL-6 levels higher in septic shock than controls. Increased levels of IL-6 were positively associated with IL-10 levels in septic shock, indicating correlation with sepsis severity	(56)
Adult	Sepsis:32 - Sepsis: 19 - Septic shock: 13 Healthy controls: 15	Blood sample was taken at initial suspicion of sepsisMean age (±SD) at sepsis:70.8 (±12.7) years	Significantly elevated IL-6 levels in septic patients compared to controls Significantly elevated levels in septic shock compared to sepsis without shock	(57)
Term(GA range 37–42 weeks)	Clinical (*n* = 10) and confirmed (*n* = 3) LOS: 13 - Sepsis: 4 - Severe sepsis: 6 - Septic shock: 3	Blood sample was taken within 24 h initial suspicion of sepsisMedian (IQR) age at sepsis:10 (7–22) days	Increased IL-6 levels are associated with septic shock	(53)
Mix of preterm andTerm(mean GA 35.8 ± 4.1)	Confirmed sepsis: 26 (EOS *n* = 13 and LOS *n* = 13) - Survivors: 17 - Non-survivors: 9 Controls: 29 Mortality: EOS deaths <2 days: 5 LOS deaths >7 days: 4 Time from sepsis onset to death not described	Blood samples were taken at sepsis evaluation before antimicrobial therapy (time 0) and on days 3 and 7 followingMean (±SD) age at sepsis:EOS 1.9 (±1.1) daysLOS 20.6 (±8.4) days	IL-6 significantly increased progressively during sepsis episode in the non-survivors IL-6 significantly decreased progressively during sepsis episode in the survivors	(55)
Mix of preterm andTerm(mean GA not described)	Confirmed sepsis: 50 (EOS *n* = 41 and LOS *n* = 9) - Survivors: 33 - Non-survivors: 17 Non-sepsis inflammation: 50 Controls: 50 Time from sepsis onset to death not described	Blood samples were taken at sepsis evaluation (time 0) and on days 1 and 2 followingAge at sepsis not described	IL-6 was significantly elevated in non-survivors compared to survivors, at time all three timepoints	(54)
**IL-8**				
Adult	Septic shock: 20 SIRS: 11 Healthy controls: 10	Blood sample was taken within 24 h initial suspicion of sepsisAge at septic shock: 68 years	IL-8 levels elevated compared to SIRS and control. Increased levels of IL-8 are positively associated with IL-10 levels in septic shock, indicating correlation with sepsis severity	(56)
Term(GA range 37–42 weeks)	Clinical (*n* = 10) and confirmed (*n* = 3) LOS: 13 - Sepsis: 4 - Severe sepsis: 6 - Septic shock: 3	Blood sample was taken at initial suspicion of sepsisMedian age at sepsis:10 (IQR 7–22) days	Increased IL-8 levels gradually increased with sepsis severity, but not significantly	(53)
Mix of preterm andTerm(mean GA 35.8 ± 4.1)	Confirmed sepsis: 26 (EOS *n* = 13 and LOS *n* = 13) - Survivors: 17 - Non-survivors: 9 Controls: 29 - Mortality: EOS deaths <2 days: 5 LOS deaths >7 days: 4 Time from sepsis onset to death not described	Blood samples were taken at sepsis evaluation before antimicrobial therapy (time 0) and on days 3 and 7Mean (±SD) age at: EOS 1.9 (±1.1) daysLOS 20.6 (±8.4) days	IL-8 increased progressively during sepsis episode in the non-survivors (only significantly between time 0 and day 3) IL-8 significantly decreased progressively during sepsis episode in the survivors	(55)
Mix of preterm andTerm(mean GA not described)	Sepsis: 50 (EOS *n* = 41 and LOS *n* = 9) - Survivors: 33 - Non-survivors:17 Non-sepsis inflammation: 50 Controls: 50 Time from sepsis onset to death not described	Blood samples were taken at sepsis evaluation (time 0) and on days 1 and 2Age at sepsis not described	IL-8 was significantly elevated in non-survivors compared to survivors, at time all three timepoints	(54)
**IL-10**				
Adult	Septic shock: 38 - Survivors: 22 - Non-survivors: 16 Mortality within 28 days after diagnosis. Time from sepsis onset to death not described	Blood samples were taken on days 1–2, 3–4, 5–7, and 8–15 days following initial suspicion of sepsisMean age at sepsis:64 years (95% CI 59–69)	IL-10 levels were significantly elevated throughout the septic episode in non-survivors compared to survivors	(52)
Adult	Infection (includes more than only sepsis): 399 - Survivors: 366 - Non-survivors: 33 Time from sepsis onset to death unclear	Blood sample was taken when empirical antibiotics commencedMedian (IQR) age at sepsis: 61 (45–77) years	IL-10 levels were significantly higher in the non-survivors. Increased IL-10 levels were associated with increased risk of mortality	(58)
Adult	Septic shock: 20 SIRS: 11 Healthy controls: 10	Blood sample was taken within 24 h initial suspicion of sepsisAge at septic shock: 68 years	IL-10 levels more elevated than controls. Increased levels of IL-6 and IL-8 are positively associated with IL-10 levels in septic shock, indicating correlation with sepsis severity	(56)
Adult	Sepsis:32 - Sepsis: 19 - Septic shock: 13 Healthy controls: 15	Blood sample was taken at time of initial suspicion of sepsisMean (±SD) age at sepsis:70.8 (±12.7) years	Significantly elevated IL-10 levels in septic patients compared to controls. Significantly elevated levels in septic shock compared to sepsis without shock	(57)
Adult	Sepsis: 61 - Survivors: 41 - Non-survivors: 20 Time from sepsis onset to death not described	Blood sample was taken on day of admission and the next dayMedian (IQR) age at sepsis in years:Survivors 52.5 (36–61.5)Non-survivors 54.5 (42.5–62.5)	Significantly elevated IL-10 levels in non-survivors compared to survivors	(59)
Adult	Post-operative sepsis: 35 - Survivors: 24 - Non-survivors: 11 Post-operative non-sepsis controls: 85 Mean time to mortality 22.3 (±6.6) days. Time from sepsis onset to death not described	Blood sample was taken at time of initial suspicion of sepsisMean (±SEM) age at sepsis:61 (±2) years	Sepsis is associated with deficient IL-10 production. Sepsis survival correlated with recovery of pro-inflammatory secretion, but not IL-10	(60)
Term(GA range 37–42 weeks)	Clinical (*n* = 10) and confirmed (*n* = 3) LOS: 13 - Sepsis: 4 - Severe sepsis: 6 - Septic shock: 3	Blood sample was taken at time of initial suspicion of sepsisMedian (IQR) age at sepsis: 10 (7–22) days	Increased IL-10 levels gradually increased are with sepsis severity, but not significantly	(53)
**IL-10/TNFα RATIO**				
Adult	Septic shock: 38 - Survivors: 22 - Non-survivors: 16 Mortality within 28 days after diagnosis. Time from sepsis onset to death not described	Blood samples were taken on days 1–2, 3–4, 5–7, and 8–15 days following initial suspicion of sepsisMean age at sepsis:64 years (95% CI 59–69)	IL-10/TNFα ratio was significantly increased during the first days of sepsis in non-survivors compared to survivors	(52)
Adult	Infection (includes more than only sepsis): 399 - Survivors: 366 - Non-survivors: 33 Time from sepsis onset to death unclear	Blood sample was taken when empirical antibiotics commencedMedian (IQR) age at sepsis: 61 (45–77) years	IL-10/TNFα ratio was significantly higher in non-survivors compared to survivors	(58)
Neonate of any GA	Not assessed	–	–	–

*GA, gestational age; LOS, late-onset sepsis; EOS, early-onset sepsis; VLBW, very low birth weight; SIRS, systemic inflammatory response syndrome; IL, interleukin; TNFα, tumour necrosis factor alpha; IFNγ, type II interferon; IQR, interquartile range; SD, standard deviation; CI, confidence interval*.

## Inflammatory cytokine secretion in response to infection in adults and neonates

### Circulating inflammatory cytokines in adult sepsis

Together, pro- and anti-inflammatory cytokines influence the innate immune responses to infection (88). Plasma levels of pro-inflammatory cytokines, including TNFα, IL-6, IL-8, IL-1β, interferon gamma (IFNγ), and the anti-inflammatory cytokines IL-10 and IL-4 are elevated in adults with sepsis and septic shock (52, 56–58, 72, 89)—study details described in Table 1. Interestingly, those with septic shock had both higher pro- and anti-inflammatory levels than patients without shock (57), with the levels of IL-6 and IL-8 positively associated with IL-10 levels (56), indicating a correlation with sepsis severity. Further support for the positive association between sepsis severity and immunosuppression are provided by reports of increased mortality in septic patients with elevated IL-10 levels or a high IL-10/TNFα ratio (52, 57–59). Additionally, patients who regained organ function by day 4 following sepsis onset had a significantly higher TNFα production capacity compared to those with ongoing organ failure by day 6 (51). While there is concomitant secretion of pro- and anti-inflammatory cytokines during sepsis, the increased ratio of IL-10 to TNFα is associated with sepsis mortality and immunosuppression (52, 58), however, the mechanism for this association has yet to be elucidated.

### Functional assessment of cytokine secretion in adult sepsis

Pro-inflammatory (TNFα, IL-1β, IL-6, IL-12) and anti-inflammatory (IL-10) responses occur concomitantly in stimulated whole blood and isolated monocytes from septic adults, albeit at a reduced capacity compared to healthy adults (60, 84, 89–91). Interestingly survival in these patients was associated with the recovery of pro-inflammatory cytokine production, but not IL-10 production—the IL-10/TNFα ratio was not reported (60). A similar pattern of decreased TNFα, IL-6, IFNγ, and IL-10 was also observed in a post-mortem study of stimulated splenocytes from patients who died of sepsis (84). These results suggest patients with sepsis have a sub-optimal capacity to produce pro- and anti-inflammatory cytokines, which is inversely associated with sepsis severity, especially when IL-10 levels remain relatively higher, eventually leading to organ failure, septic shock, and/or death.

### Circulating inflammatory cytokines in neonatal sepsis

In both preterm and term neonates with EOS or LOS, the circulating levels of pro-inflammatory cytokines, IL-6 (28, 53–55, 92–96), IL-8 (53–55), and IFNγ (92, 94, 95) are consistently elevated compared to non-septic neonates. Whereas, TNFα and IL-1β levels are more variable (28, 53, 95, 96) or increased (28, 53–55, 92, 94, 96). The inconsistent reports of TNFα and IL-1β concentrations in neonatal sepsis may be a confounded by the kinetics and short half-life of circulating TNFα and IL-1β and the timing of sample collection relative to the onset of sepsis (97, 98). Circulating anti-inflammatory IL-10 concentrations (28, 53, 92, 94, 95) are elevated in preterm and term neonates with EOS or LOS compared to non-septic neonates, whereas the concentration of IL-4 is more variable (28, 92, 94, 95). These studies did not report the ratio of IL-10 to TNFα. Neonatal and sepsis characteristics, and relevant outcomes of these studies not in relation to sepsis severity are described in the Supplementary Table 1.

Gestational age may significantly influence the neonatal cytokine response to infection. In 14 very preterm (mean GA 28.7 ± 1.3 weeks) and 12 moderately preterm (mean GA 34.6 ± 1.8 weeks) neonates with confirmed or clinical sepsis (including LOS and EOS), the cytokine profiles differed (92). During sepsis, the levels of IFNγ, IL-6, IL-10, and IL-4 were significantly elevated in the moderate preterm group only. In contrast, the levels of TNFα did not significantly change from pre-sepsis to during sepsis in either group. These results suggest that increasing GA may be associated with a more robust pro- and anti-inflammatory response. While the lack of inflammatory response in very preterm infants may explain the increased incidence and severity of sepsis (99). The results from this small study do not allow firm conclusions on neonatal clinical outcomes.

Increased IL-6, IL-1β, and IL-8 cytokine production might be associated with sepsis severity and/or mortality in neonates with sepsis (53–55)—study details described in Table 1. Silveira-Lessa and colleagues investigated cytokine production in 13 term (GA range 37–42 weeks) neonates with confirmed (*n* = 3) and clinical (*n* = 10) LOS, including 6 with severe sepsis [classified as per the international paediatric consensus definition for sepsis (39)] and 3 with septic shock (53). Higher IL-6 and IL-1β levels were significantly associated with septic shock (*n* = 3) and mortality (*n* = 2), respectively (53). Increased levels of IL-8 and IL-10 were associated with sepsis, whereas TNFα was not changed. The sample size in this study was small, thus limiting the interpretation of significant changes in cytokine levels associated with sepsis severity. Similarly, increased levels of IL-6 and IL-8 persisted for longer in preterm and term neonates with fatal LOS and EOS (combined *n* = 26), whereas the duration of elevated TNFα levels was variable (54, 55). Similar to adults (100, 101), it has been suggested that IL-6 concentrations are a strong indicator of sepsis prognosis in neonates (53, 54). The results from these studies, summarised in Table 1, suggest increased and persistent levels of pro-inflammatory mediators correlate with greater neonatal sepsis severity. However, evidence as to the association between persistent anti-inflammatory mediator levels and clinical sepsis severity in neonates remains inconclusive.

The focus of the above neonatal studies was to characterise patterns of cytokine production in septic neonates as potential predictive or diagnostic tools or markers of development, and not necessarily to associate cytokine responses to clinical outcomes. From these results, we can acknowledge that neonates are capable of eliciting a cytokine response similar to that of adults in response to infection (52, 56–58, 72, 89). We cannot, however, infer that the pattern of cytokine production associated with SII and sepsis severity in adults, is also present in these neonates.

### Functional assessment of cytokine secretion in neonatal sepsis

One study has assessed monocyte cytokine production in 32 extremely (median gestation 25.5 weeks, range <28 weeks) and 44 very (median gestation 29 weeks, range 28–32 weeks) preterm neonates with confirmed (*n* = 38) and clinical (*n* = 38) LOS (102). The authors of this report found that following monocyte stimulation with Pam3Cys, both groups produced equivalent IL-1β, but extremely preterm neonates produced higher IL-18 (102). These results highlight the influence GA has on neonatal immune regulation, but outcomes are limited. There is a need to further investigate if immune cell dysfunction at the time of sepsis underpins immunosuppression in neonatal sepsis.

## Reduced MHC class II expression in adult and neonatal sepsis

### HLA-DR expression in adult sepsis

The upregulation of HLA-DR cell surface expression on APCs is a hallmark of APC activation and essential for increased presentation of antigens to naïve T cells, a critical step for initiating the adaptive immune response (103). Low HLA-DR expression associated with SII is often referred to as immunoparalysis (6, 104, 105) and the established cut-off for identifying immunoparalysis in adult patients with sepsis is <30% HLA-DR positive monocytes (6, 106, 107). In adults, sepsis and septic shock have been shown to negatively affect HLA-DR cell surface expression and cause immunosuppression (52, 61–63, 73, 76, 108)—study details summarised in Table 2. Low HLA-DR expression on monocytes and immunoparalysis are related to sepsis severity as shown by a significant increase in SOFA scores in adults with sepsis (61, 62). Monocyte HLA-DR expression is also significantly lower in sepsis non-survivors compared to survivors (52, 59, 61, 63).

**Table 2:** Sepsis-induced immunosuppression—association of monocyte surface HLA-DR expression with sepsis severity in neonates and adults with sepsis.

**Adult or neonatal GA**	**Cohort sepsis characteristics (*n*) and mortality (if applicable)**	**Time of blood sampling and age at sepsis**	**Observation in septic cohort**	**References**
Adult	Septic shock: 38 - Survivors: 22 - Non-survivors: 16 Mortality within 28 days after diagnosis. Time from sepsis onset to death not described	Blood samples were taken on days 1–2, 3–4, 5–7, and 8–15 days following initial suspicion of sepsisMean age at sepsis:64 years (95% CI 59–69)	Decreased % HLA-DR expression in septic shock Significantly lower % HLA-DR expression in non-survivors compared to survivors	(52)
Adult	Sepsis: 61 - Survivors: 41 - Non-survivors: 20 Time from sepsis onset to death not described	Blood sample was taken on day of admission and the next dayMedian (IQR) age at sepsis in years:Survivors 52.5 (36–61.5)Non-survivors 54.5 (42.5–62.5)	Decreased HLA-DR expression in sepsis. Significantly lower in non-survivors compared to survivors	(59)
Adult	Organ dysfunction during sepsis: 37 SIRS: 13 Healthy control: 20	Blood sample was taken within 24 h of sepsis developmentMedian (IQR) age at sepsis:69.4 (±2.7) years	Progressive significant decrease in CD14/HLA-DR expression in the organ dysfunction during sepsis group	(61)
Adult	Sepsis/septic shock: 20 Post-surgical inflammation: 20 Non-sepsis controls: 10	Blood sample was taken within 24 h of study inclusionMedian (IQR) age at sepsis:60 (53–67) years	Decreased HLA-DR surface protein and mRNA expression in sepsis/septic shock TNFα:HLA-DR ratio correlates negatively with SOFA score	(62)
Adult	Sepsis: 17 - Survivor: 6 - Non-survivors: 11 Non-sepsis controls: 10 Healthy control: 12 Time to mortality: During 1^st^ septic episode *n* = 9 During 2^nd^ septic episode *n* = 2 Time from sepsis onset to death not described	Blood sample was taken upon admission to the studyMean (±SEM) age at sepsis:71 (±5) years	HLA-DR expression significantly decreased in sepsis group. HLA-DR expression was significantly lower in non-survivors, compared to survivors 6 of 17 with sepsis later developed nosocomial infections	(63)
Mix of preterm andTerm(mean GA 37.5 ± 3.8)	Clinical (*n* = 22) and confirmed (*n* = 18) LOS: 40 - Survivor: 32 - Non-survivor: 8 Non-sepsis disorder: 24 Controls: 25 Time to mortality: during hospital stay. Time from sepsis onset to death not described	Sample collection time not describedMean (±SD) age at sepsis:16.3 (±5.8) days	Significantly lower HLA-DR expression in sepsis group HLA-DR expression was significantly lower in non-survivors compared to survivors No significant difference HLA-DR expression between term and preterm No significant difference HLA-DR expression between clinical and confirmed LOS	(64)
Mix of moderate preterm and term (median GA 36; IQR 32–39 wks)	Clinical (*n* = 42) and confirmed (*n* = 21) EOS and LOS: 63 -Survivor: 50 -Non-survivor: 13 Non-sepsis: 37 Controls: 29 Mortality <30 days *n* = 13 Time from sepsis onset to death not described	Blood sample taken upon initial suspicion of sepsisMedian (IQR) age at sepsis:4 (2–11) days	HLA-DR expression was significantly decreased in the sepsis group. Lower, but not significantly, in non-survivors compared to survivors	(65)
Preterm (mean GA 31 ± 2 weeks)	EOS: 22 - Mild sepsis: not described -Severe sepsis: not described Controls: Not described	Blood samples taken at admission to NICU during first 48 h of life, during infection, and recoveryMean age at sepsis: Not described	Percent of HLA-DR positive monocytes significantly recovered in those with mild sepsis. Percent expression of HLA-DR on monocytes significantly dropped followed by a significant recovery in those with severe sepsis	(66)^*^

*HLA-DR, Human Leukocyte Antigen-DR isotype; GA, gestational age; LOS, late-onset sepsis; EOS, early-onset sepsis; VLBW, very low birth weight; SIRS, systemic inflammatory response syndrome; SD, standard deviation; IQR, inter-quartile range*.

* *Conference abstract only, limited data available*.

Low HLA-DR expression on adult monocytes during sepsis is associated with altered immune responses, including imbalanced secretion of pro- and anti-inflammatory mediators and reduced antigen presentation capacity, and importantly sepsis severity and mortality (52, 59, 61–63). Decreased monocyte HLA-DR expression in critically ill adults with sepsis or septic shock has also been associated with a prominent shift toward significantly increased circulating levels of IL-10 (52, 59, 63). Interestingly, IL-10 mediates HLA-DR expression on monocytes (109–111), suggesting that HLA-DR expression could be a marker of SII-related cytokine changes.

In a study of 17 critically ill adults with sepsis, decreased expression of HLA-DR and CD86 on monocytes and CD28 on lymphocytes was significantly associated with reduced antigen presentation (63). Although the authors did not find any association between the levels of HLA-DR expression or antigen presentation and development of secondary infections, 6 of the 8 patients who survived sepsis went on to develop a secondary infection, 2 of whom later died (63).

### Low HLA-DR expression in neonatal sepsis

Several studies reported a decrease in monocyte HLA-DR expression in preterm and term neonates with confirmed or clinical sepsis (including EOS and LOS) (64–66, 93, 112). Neonatal and sepsis characteristics, and relevant outcomes of these studies not in relation to sepsis severity are described in the Supplementary Table 2. Decreased HLA-DR expression observed in mixed cohorts of preterm and term with neonatal sepsis appears unrelated to the GA (64, 93, 112). Serial assessment of HLA-DR expression during neonatal sepsis demonstrated that 3 days after sepsis onset, HLA-DR expression in both preterm and term neonates were similar to those without sepsis (93). However, low HLA-DR expression is a possible marker of sepsis-related mortality, as monocyte HLA-DR expression is down-regulated in term and preterm non-survivors of sepsis compared to survivors (64, 65)—study details summarised in Table 2. In this small study, Genel et al reported a significant decrease in monocyte HLA-DR expression between non-survivor (*n* = 8) and survivor (*n* = 32) preterm and term (median GA 36 weeks) neonates with confirmed (*n* = 18) and clinical (*n* = 22) LOS (mean postnatal age 16.3 days) (64). The preterm and term neonates with ≤30% HLA-DR positive monocytes had a 30-fold higher risk of mortality (Odds ratio 30); with 53.8% mortality among those with ≤30% HLA-DR positive monocytes compared to only 3.7% in neonates with >30% HLA-DR positive monocytes (64), similar to adults with confirmed immunoparalysis (61). Unlike for HLA-DR surface expression levels, the proportion of cells expressing any HLA-DR in neonates is correlated with GA and acts as a predisposing factor for sepsis as reported in 31 very low birth weight infants (VLBW; GA range 23–31 weeks) with clinical (*n* = 14) and confirmed (*n* = 17) sepsis (EOS *n* = 2 and LOS *n* = 29) (113). Pradhan et al. suggested that monocyte HLA-DR expression, combined with CD64 expression on neutrophils, may be a useful prognostic marker for neonatal sepsis (65).

There is a decrease in HLA-DR positive monocytes among preterm and term neonates with sepsis, compared to non-septic neonates (64, 66, 93, 112, 114, 115). Decreased HLA-DR positive monocytes in neonates with sepsis appears unrelated to the GA (64, 93, 112). Fotopoulos et al. monitored the proportion of HLA-DR positive monocytes over the course of a septic episode in preterm neonates (mean GA 31 weeks), with and without EOS (66). They reported that the percentage of HLA-DR positive monocytes significantly recovered over the course of sepsis in those neonates with mild sepsis, while those with severe sepsis showed a significant drop followed by a rise only upon recovery (66). While the authors did not provide the criteria for defining sepsis severity, this data may suggest that a decreased percentage of HLA-DR positive monocytes is associated with sepsis severity in neonates, however additional research in this area is essential.

## Expansion of immunosuppressive cells in adult and neonatal sepsis

### Immature neutrophils in adult sepsis

The ability of immature neutrophils to suppress T cell proliferation was first observed by Pillay et al. (116), although the mechanism for suppression remains unclear. An increased frequency of immature neutrophils has been observed in adults with sepsis and is associated with sepsis severity, poor clinical outcomes, and increased risk of septic shock and mortality (67–69)—study details summarised in Table 3. As sepsis becomes more severe in adults, the increased frequency in immature neutrophil has been shown to be associated with a decrease in T cell proliferation (67).

**Table 3:** Sepsis-induced immunosuppression—association of immunosuppressive cell expansion with sepsis severity in neonates and adults with sepsis.

**Adult or neonatal GA**	**Cohort sepsis characteristics (*n*) and mortality (if applicable)**	**Time of blood sampling and age at sepsis**	**Observation in septic cohort**	**References**
**IMMATURE NEUTROPHILS**				
Adult	Sepsis: 177 - Sepsis: 82 - Organ dysfunction during sepsis: 66 - Septic shock: 29 Outpatient control: 50 Community-acquired infection without SIRS: 15	Blood sampling was done as part of routine haematological analysis. Sample collection time not describedMean (±SD) age at sepsis:Sepsis: 57 (±22) yearsOrgan dysfunction during sepsis: 62 (±17) yearsSeptic shock: 63 (±14) years	Sepsis group had increased immature granulocytes compared to the two control groups	(67)
Adult	Sepsis: 83 - Confirmed sepsis: 51 - Clinical sepsis: 32 Non-infection SIRS: 39 Non-SIRS: 14 Healthy control: 20	Blood sample was taken within 48 h of admission to the intensive care unitMean (±SD) age at sepsis:Confirmed sepsis: 62 (±16) yearsClinical sepsis: 66 (±13) years	Immature neutrophils were elevated in the sepsis group. Immature neutrophils frequency was significantly higher in confirmed sepsis compared to clinical sepsis and non-infection inflammation	(68)
Adult	Septic shock: 43 - Survivor: 35 - Non-survivors: 8 Healthy controls: 23 Time to mortality: within 28 days of sepsis onset. Time from sepsis onset to death not described	Blood samples were taken at days 3–4 and 6–8 after onset of septic shockMedian (IQR) age at septic shock in years: 70 (65–80)	Increased circulating immature granulocytes associated with increased risk of death	(69)
Mix of:Preterm ≤ 28 weeks GA (*n* = 21)Preterm>28–36 weeks GA(*n* = 123)Term>36 weeks GA (*n* = 141)	Clinical and confirmed EOS (*n* = 76) and LOS (*n* = 134): 210 - Survivor: 222 - Non-survivor: 63 No sepsis: 75 Time from sepsis onset to death not described	Blood sample was taken upon initial suspicion of sepsisMean (±SD) age at sepsis:6.7 (±7.4) days	Severity of neutrophil left shift correlates with increased sepsis mortality risk in both preterm and term neonates	(70)
VLBW < 1500g(approximate mean GA 27 weeks)	EOS: 5 - Survivor: 0 - Non-survivors: 5 LOS: 15 - Survivor: 0 - Non-survivors: 15 Controls: NA Mean (±SD) age of death: EOS:1.6 (±0.5) days LOS:17.8 (±12.1) days Time from sepsis onset to death not described	Post-mortem examination completed within 2 h of deathMean (±SD) age at sepsis:EOS: 0 (±0) daysLOS: 14.1 (±9.9) days	EOS: Slightly elevated, but not significantly, circulating immature neutrophils during early phase of sepsis LOS: Elevated circulating immature neutrophils. Significantly elevated during terminal stages	(71)
**T REGULATORY CELLS**				
Adult	Sepsis: 80 - Sepsis: 31 - Organ dysfunction during sepsis: 33 - Septic shock: 16 Healthy controls: 18	Blood sample was taken within 24 h after sepsis diagnosisMedian (IQR) age at sepsis:Sepsis: 45 (28–72) yearsOrgan dysfunction during sepsis: 54 (18–87) yearsSeptic shock: 64 (18–84) years	Increased Treg mRNA in sepsis patients	(72)
Adult	Sepsis: 32 - Sepsis:19 - Septic shock: 13 Healthy controls: 10	Blood sample was taken at time of sepsis diagnosisMean (±SD) age at sepsis:70.8 (±12.7) years	Significantly increased Tregs in CD4^+^ T cells in sepsis group. Significantly higher in septic shock than sepsis without shock	(57)
Adult	Septic shock: 16 - Survivor: 7 - Non-survivor: 9 Healthy controls: 36	Blood sampling was taken on days 1, 3, 5 and 7–10 following sepsis onsetMean age at sepsis: 54 years	Elevated circulating CD4^+^ Treg cells in the sepsis group. CD4^+^ Treg more elevated in non-survivors compared to survivors	(73)
Adult	Sepsis: 118 - Sepsis: 78 - Septic shock: 40 Healthy control: 21	Blood sample was taken the day of study inclusionMedian (IQR) age at:Sepsis: 73.5 (62–81) yearsSeptic shock: 78.5 (60–84) years	Increased Tregs in CD4^+^ T cells in the sepsis group	(74)
Adult	Sepsis: 42 - Survivor: 23 - Non-survivor: 19 Healthy control: 14 Time to mortality: < 28 days. Time from sepsis onset to death not described	Blood samples were taken days 0 and day 5Mean (±SD) age at sepsis:49.1 (±10.2) years	Increased CD39+ Tregs in the sepsis group. Higher Treg expression in those with organ failure and non-survivors	(75)
Neonate of any GA	Not assessed	–	–	–
**MYELOID DERIVED SUPPRESSOR CELLS**				
Adult	Sepsis: 94 - Organ dysfunction during sepsis: 22- Septic shock: 72 Non-septic ICU: 11 Healthy controls: 67	Blood sample taken within 3 days of sepsis diagnosisMedian (IQR) age, in years, at:Organ dysfunction during sepsis: 57 (41–75)Septic shock: 63 (53–73)	In the sepsis group MDSC genes are up-regulated, G-MDSCs expanded and plasma MDSC mediator levels are increased	(76)
Adult	Septic shock: 74 Healthy controls: 18	Blood samples were taken within 12 h of sepsis diagnosis, and on days 1, 4, 7, 14, 21 and 28Mean age at sepsis: 60 years	MDSCs persistently increased in the septic shock group. MDSCs were functionally immunosuppressive	(77)
Adult	Sepsis: 24 - Sepsis: 12 - Septic shock: 12 Non-sepsis: 12	Blood samples were taken at enrolment, and on days 2–4 and 7-dischargeMedian (IQR) age at:Sepsis: 45 (39–55) yearsSeptic shock: 52 (45–57) years	G-MDSCs were increased in the sepsis group. G-MDSCs were significantly higher in septic shock compared to sepsis without shock. G-MDSCs were functionally immunosuppressive	(78)
Neonate of any GA	Not assessed	–	–	–

*GA, gestational age; LOS, late-onset sepsis; EOS, early-onset sepsis; VLBW, very low birth weight; ICU, intensive care unit; Treg, T regulatory cells; MDSC, myeloid derived suppressor cells; G-MDSC, granulocytic-myeloid derived suppressor cells; SIRS, systemic inflammatory response syndrome; SD, standard deviation; IQR, inter-quartile range*.

### Immature neutrophils in neonatal sepsis

While neonates with sepsis have increased numbers of circulating immature neutrophils compared to neonates without sepsis, this is not a reliable diagnostic marker (117–121). There is a paucity of data on whether the T cell suppressive function of immature neutrophils contributes to sepsis severity, adverse outcomes, and increased mortality in neonatal sepsis. Saied and colleagues evaluated neutrophil left shift for its predictive value in sepsis outcomes in extremely preterm (*n* = 21; GA range ≤ 28 weeks), very/moderate preterm (*n* = 123; GA range >28–36 weeks), and term (*n* = 141; GA range >36 weeks) neonates with confirmed or clinical sepsis (EOS *n* = 76; LOS *n* = 134) (70). Although T cell function was not assessed in this study, they found that an increase in left shift (and hence proportions of immature neutrophils) correlates with increased sepsis mortality risk in preterm and term neonates (70). Further to this, Itoh et al. found a high number of circulating immature neutrophils, in addition to depleted lymphocytes in the thymus and hypertrophic spleen, in 15 VLBW infants (<1,500 g) that died from confirmed LOS (mean time from sepsis onset to death 3.7 ± 3.3 days) (71). Yet, the number of circulating immature neutrophils was only slightly elevated during the initial stage of sepsis the neonates that died from confirmed EOS (*n* = 5; mean time from sepsis onset to death 1.6 ± 0.5 days) (71). However, the number of neonates with sepsis were low and a non-septic control group was lacking for comparison. The frequency of immature neutrophils and its relation to sepsis severity observed in both neonates and adults are summarised in Table 3. These two studies suggest that immature neutrophils may be associated with worse outcomes with neonatal sepsis, however, further studies with larger sample size and non-septic controls are essential. Whether increased numbers of immature neutrophils are a consequence of sepsis or whether they cause more severe infection due to their immunosuppressive function on T cells remains to be determined in neonatal sepsis.

### Regulatory T cells (tregs) in adult sepsis

Tregs play an important role in the maintenance of immune homeostasis, however their role in immunosuppression during sepsis is not entirely clear (7, 122, 123). Several studies have reported an elevated proportion of Tregs following the onset of sepsis or septic shock in adults, and associated this with an increased risk of immunosuppression, mortality, and morbidity (57, 72–75)—study details summarised in Table 3. The increased risk of mortality associated with sepsis and sepsis shock may be attributed to the immunosuppressive functions of Tregs by: (a) directly inhibiting effector CD4+ T cell proliferation and cytokine secretion (124–126); (b) indirectly supressing APC/T-cell receptor mediated CD4+ and CD8+ T cell activation (125–127); (c) suppressing T cell activation through increased expression of programmed cell death-1 (PD-1) receptor (128); or (d) suppressing other immune effector cells such as natural killer cells, B cells, and monocytes (129–131).

### Regulatory T cells (tregs) in neonatal sepsis

Identifying the role of Tregs in neonatal sepsis is an emerging area of research. The proportion of Tregs is elevated in term neonates with confirmed sepsis (*n* = 30) (132). Likewise, the proportion of Tregs is higher in 22 preterm neonates (mean GA 28.1 ± 3.7 weeks) with clinical EOS (133). Sepsis severity and mortality were not discussed in these studies, nor was the age of sepsis onset. Surprisingly, unlike cord blood (134–137), the elevated proportion of Tregs reported in septic neonates may not be affected by GA (133), suggesting sepsis alone, and not gestation, influences Treg frequency, postnatally. There are no data on the potential impact of Tregs on sepsis severity, immunosuppression, or mortality during neonatal sepsis. Treg frequencies and further functional analysis is required to determine whether Tregs suppress T cell proliferation and function during neonatal sepsis, as observed in adults with SII (74, 122, 123, 125).

### Myeloid-derived suppressor cells (MDSCs) in adult sepsis

In healthy adults, the immature cells of the myeloid lineage, namely monocytic- and granulocytic-MDSCs, rapidly differentiate into DCs, macrophages, and granulocytes and act to preserve innate immunity (138). The expansion of MDSCs to suppress both the innate and adaptive immune responses is a phenomenon under investigation in adults with SII (77, 138). MDSC expansion has been observed in adults with sepsis and septic shock (76–78, 138), as summarised in Table 3. The immune suppressive characteristics of MDSC expansion, namely inhibition of T cell proliferation (76–78, 138) and increased secretion of immunosuppressive mediator IL-10 (78, 138), have been observed in adults with sepsis. MDSC expansion is associated with sepsis severity (77, 78) and adverse outcomes, such as chronic immune suppression following prolonged MDSC expansion in critically ill adults with sepsis (77). Expansion of MDSCs has also been shown to be associated with higher risk for subsequent nosocomial infections (76, 77), a characteristic found among patients with SII. However, the frequency of MDSCs among sepsis survivors and non-survivors was found to be similar, suggesting MDSC expansion alone does not influence mortality (76).

### Myeloid-derived suppressor cells (MDSCs) in neonatal sepsis

Only one group has investigated the frequency of MDSCs during neonatal sepsis, and found a significant increase in the frequency of MDSCs in 10 preterm neonates (mean GA 25 ± 3 weeks) with early- and late-onset clinical (80%) and confirmed (20%) sepsis compared to preterm neonates (GA range 23 to < 37 weeks) without sepsis (139). No associations with severity or sepsis outcomes were made in this study. With the limited data and small sample size in this publication the characterisation of MDSCs during neonatal sepsis requires further evaluation.

## Prolonged immunosuppression and alarmins

Alarmins (also referred to as damage-associated molecular patterns), such as S100A8 and S100A9, are pro-inflammatory mediators present in low levels in circulating myeloid cells, namely monocytes and granulocytes, even in healthy subjects. S100A8 and S1009A are up-regulated in response to bacterial products, as well as pro- (e.g., TNFα and IL-1β) and anti-inflammatory cytokines (e.g., IL-10 and transforming growth factor β) (140). Up-regulation of S100A8 and S100A9 positively regulates MDSC frequency and function (141, 142), stimulates Treg expansion (140), and induces endotoxin tolerance by rendering phagocytes unresponsive to secondary Toll-Like Receptor-4 stimulation (143). Whether S100A8 and S100A9 function to enhance inflammation or to support immunosuppression is still unclear.

### Alarmins in adult sepsis

Plasma levels of S100A8 and S100A9 were elevated, as was up-regulation of S100A12, S100A9, and arginase-1 gene expression, in adults with sepsis, compared to non-septic patients in intensive care (76). The increased levels of S100A12, S100A9, and arginase-1 were associated with MDSC expansion, and high initial levels of granulocytic-MDSCs, arginase-1 and S100A12 were associated with subsequent infections (76). Alarmins, such as the S100 proteins, may therefore, play multiple roles in adult SII.

### Alarmins in neonatal sepsis

S100A8/A9 levels were elevated in eight neonates with confirmed sepsis (144). Gestational age, postnatal age at sepsis onset and relation of alarmin levels to the severity of the sepsis were not discussed. Whether alarmins, such as S100A8/S100A9, play a role in MDSC expansion and immunosuppression in neonatal sepsis requires further investigation.

## Compromised T cell effector cell function in adult and neonatal sepsis

### Immune checkpoint molecule expression in adult sepsis

The increased expression of the negative co-stimulatory molecule PD-1, and its associated ligand (PD-L1), on circulating monocytes, neutrophils and effector T cells may contribute to SII (79). Increased expression of PD-1/PD-L1 on monocytes and lymphocytes is associated with decreased monocyte HLA-DR expression, increased proportions of Tregs, and T cell exhaustion (3, 4, 79, 145–147). Several groups have reported an over-expression of PD-1 on T cells, including CD4+ and Tregs, and PD-L1 on monocytes in adults with sepsis and septic shock compared to healthy adults (74, 79, 80, 83, 84). Increased expression of PD-1 and PD-L1 on lymphocytes and monocytes is associated with more organ dysfunction during sepsis and increased risk of secondary infections and mortality (74, 79, 80), as summarised in Table 4. In addition, increased PD-L1 expression on monocytes has been shown to be an independent predictor of mortality in septic shock patients (80).

**Table 4:** Sepsis-induced immunosuppression—association of effector cell function and programmed cell death-1 receptor expression with sepsis severity in neonates and adults with sepsis.

**Adult or neonatal GA**	**Cohort sepsis characteristics (*n*) and mortality (if applicable)**	**Time of blood sampling and age at sepsis**	**Observation in septic cohort**	**References**
Adult	Sepsis: 118 - Sepsis: 78 - Septic shock: 40 Healthy control: 21	Blood sample was taken on day of study inclusionMedian (IQR) age at:Sepsis: 73.5 (62–81) yearsSeptic shock: 78.5 (60–84) years	Increased PD-1 expression on Tregs in sepsis group	(74)
Adult	Septic shock: 64 Trauma control: 13 Healthy control: 49	Blood samples were taken on days 1–2, 3–5, and 6–10 after diagnosisMedian (IQR) age at septic shock: 64 (54–73) years	Increased PD-1, PD-L1 expression on monocytes, and CD4^+^ T cells in septic shock group	(79)
Adult	Sepsis: 135 - Sepsis: 59 - Septic shock: 76 Healthy control: 29	Blood samples were taken 3–4 days after onset of symptomsMedian (IQR) age at:Sepsis: 71 (66–78) yearsSeptic shock: 71 (61–78) years	Increased PD-L1 expression on monocytes in the sepsis group	(80)
VLBW < 1,500 g and ≤ 32 weeks GA (mean GA 26.8 weeks)	LOS: 39 - Sepsis: 28 - Septic shock: 5 Non-survivors: 6 Control: NA Time to mortality during hospitalisation Time from sepsis onset to death not described	Blood sample was taken within 24 h of symptom onsetAge at sepsis not described	Increased PD-L1 expression on monocytes in sepsis group. Significant increases in those with septic shock and/or death compared to survivors of sepsis without shock	(81)

*GA, gestational age; LOS, late-onset sepsis; VLBW, very low birth weight; PD-1, programmed cell death protein-1; PD-L1, programmed cell death ligand-1; Tregs, T regulatory cells; SD, standard deviation; IQR, inter-quartile range*.

PD-1/PD-1L are inhibitory immune checkpoint molecules and blockade of their function to interact with other immune cells is being explored as a therapeutic agent for reversing the effects of immunosuppression (148). Pre-clinical models of sepsis, have shown that blockade of the PD-1/PD-L1 pathway with an antagonistic anti-PD-L1 antibody improves survival by inhibiting lymphocyte apoptosis and T cell exhaustion (145, 149, 150). Further to this, *in vitro* blockade of the PD-1/PD-L1 pathway in the blood from septic adults, decreases lymphocyte apoptosis, increases pro-inflammatory cytokine production and decreases IL-10 production (83, 146). Antibody blockade of PD-1 or PD-L1 as an immunomodulatory therapy for reversing immunosuppression is being trialled to improve survival in human patients with cancer (151). This promising therapy may spark exploration for PD-1 blockade immunotherapy in sepsis.

### Immune checkpoint molecule expression in neonatal sepsis

Despite the interest in exploring PD-1 blockade for reversing immunosuppression in septic adults, there is a paucity of data pertaining to PD-1 and PD-L1 expression or T cell exhaustion in neonates, and importantly neonates with sepsis. PD-1 expression was increased in 34 VLBW (<1,500 g and GA range ≤ 32 weeks) with confirmed LOS, and expression was significantly increased in 5 preterm infants with septic shock (identified using the international paediatric consensus criteria) and/or mortality (*n* = 6) compared to surviving preterm infants without shock (81). The role of GA on PD-1 expression during neonatal sepsis has not been explored. The results from this study, summarised in Table 4, suggest increased PD-1 expression may have an immunosuppressive function in neonatal sepsis, however with the limited available data this interpretation remains inconclusive.

Interestingly, Young and colleagues recently investigated the role of PD-1 in murine neonates and found improved survival in septic PD-1 knockout mice, further supporting the functional importance of PD-1 in neonatal sepsis and related mortality (152). The therapeutic potential for targeted blockade of PD-1 means that this is an area that deserves urgent exploration.

Up-regulation of carcinoembryonic antigen-related cell-adhesion molecule 1 (CEACAM1), another inhibitory immune checkpoint molecule (153), on T cells leads to reduced proliferation and cytokine secretion causing T cell suppression and subsequent prolonged immunosuppression (154, 155). Although not reported in adults with SII, the percentage of CEACAM1-positive CD4+ T cells in 12 preterm neonates with LOS is increased compared to 16 non-septic controls (155). With the small sample size and limited available data it is inconclusive as to whether increased expression of CEACAM1 on CD4+ T cells contributes to immunosuppression in septic neonates and thus requires further investigation.

## Sepsis-induced immune cell apoptosis in adults and neonates

### Immune cell apoptosis in adult sepsis

Cell death is an important step for resolving infection and maintaining immune homeostasis. However, sepsis-induced immune cell apoptosis, resulting in an overwhelming depletion of immune cells, including T cells (CD4+ and CD8+), B cells, and DCs, is evident in prospective studies of adults with sepsis (82, 83). Similar findings have been reported in post-mortem studies of adults who died from sepsis, septic shock, and sepsis-related multiple organ dysfunction (84, 85, 156, 157)—study details summarised in Table 5. The degree of immune cell apoptosis has been shown to be correlated with sepsis severity, supporting a role for apoptosis in SII (82). In support of this concept, in mice *in vivo* prevention of cell death improves sepsis survival (158–162).

**Table 5:** Sepsis-induced immunosuppression—association of sepsis-induced immune cell apoptosis and depletion with sepsis severity in neonates and adults with sepsis.

**Adult or neonatal GA**	**Cohort sepsis characteristics (*n*) and mortality (if applicable)**	**Time of blood sampling and age at sepsis**	**Observation in septic cohort**	**References**
Adult	Prospective study: Sepsis: 71 Non-sepsis:55 Healthy control: 6	Blood samples were collected at various times during sepsisMean age range at sepsis: 57–59	Increased T cell, B cell, and dendritic cell apoptosis in the sepsis group	(82)
Adult	Prospective study: Septic shock: 19 Healthy control: 22	Blood sample was collected at time of study inclusionMean (±SD) age at sepsis:58 (±4) years	Marked increase in apoptosis of CD4^+^ and CD8^+^ T cells and B cells in the septic shock group	(83)
Adult	Post-mortem study: Organ dysfunction during sepsis: 40 Trauma control: 29 Median (range) days of sepsis: 4 (1–40). Time from sepsis onset to death not described	Post-mortem sample collection occurred 30–180 min following deathMean (±SD) age at organ dysfunction during sepsis: 71.7 (±15.9) years	Extensive depletion of splenic CD4^+^ and CD8^+^ T cells and HLA-DR cells in the organ dysfunction during sepsis group	(84)
Adult	Prospective and post-mortem study Sepsis: 27 - Survivor: 2 - Non-survivors: 25 Non-septic critically ill: 16 Trauma control: 25 Mean age of death and time from sepsis onset to death not described	Sample collection was either intraoperatively (survivors) or post-mortem (15 min to 6 h following death)Mean age as sepsis not described	Depletion of splenic CD4^+^ T helper cells and B cells in the sepsis group	(85)
VLBW < 1,500 g(approximate mean GA 27 weeks)	EOS: 5 - Survivor: 0 - Non-survivors: 5 LOS: 15 - Survivor: 0 - Non-survivors: 15 Controls: NA Mean (±SD) age of death: EOS:1.6 (±0.5) days LOS:17.8 (±12.1) days Time from sepsis onset to death not described	Post-mortem examination completed within 2 h of deathMean (±SD) age at sepsis:EOS: 0 (±0) daysLOS: 14.1 (±9.9) days	EOS: No cell depletion LOS: Depletion of thymus lymphocytes	(71)
Moderate preterm(GA range 35–37 weeks)	Sepsis: 6 - Survivor: 0 - Non-survivor:6 Control mortality: 6 Mean age of death and time from sepsis onset to death not described	Post-mortem examination time not describedAge at sepsis not described	Depletion of neutrophils in the sepsis group	(86)
Mix of preterm and term(GA mean 29.2 (range 24–38) weeks)	EOS: 10 - Survivor: 0 - Non-survivor: 10 Control mortality: 20 Time to mortality within 48 h after birth. Time from sepsis onset to death not described	Post-mortem examination occurred between 4 and 12 h following deathAge at sepsis < 48 h after birth	Depletion of T cells and B cells	(87)

*GA, gestational age; LOS, late-onset sepsis; EOS, early-onset sepsis; VLBW, very low birth weight; HLA-DR, Human Leukocyte Antigen-DR isotype*.

### Immune cell apoptosis in neonatal sepsis

Three post-mortem studies have reported lymphocyte depletion in the spleen, thymus, and bone marrow in both preterm and term neonates that died from EOS and LOS compared to neonates that died of causes other than sepsis (71, 86, 87). The sample size in all three studies was small, with 5–15 neonates in the sepsis groups. There were conflicting results between the two studies that reported on lymphocyte depletion following EOS (71, 87). Only two of the three studies described the time from sepsis onset to death; death occurred within 48 h after sepsis onset for EOS (71, 87) and, on average, 3.7 (± 3.3) days following sepsis onset for LOS (71). These results, summarised in Table 5, suggest both term and preterm neonates with severe sepsis may develop sepsis-induced immune cell apoptosis, however there are no prospective studies to support this conclusion. Sepsis-induced immune cell apoptosis in relation to disease severity has not been assessed in neonates. A mouse model of neonatal sepsis found that blocking necroptosis, programmed cell death triggered by the caspase-independent pathway through death receptors, by inhibition of receptor-interacting protein kinase 1 with necrostatin-1, reduced lung injury associated with sepsis and improved survival (162).

## Is sepsis-induced immunosuppression a feature of neonatal sepsis?

In adults, SII is associated with increased risk of multi-organ failure and mortality as well as susceptibility to secondary viral and bacterial infections (3, 4, 6, 8). Similar clinical characteristics, including increased risk of multi-organ failure and mortality, are observed in neonatal sepsis (31, 163–165). It is unclear if these adverse outcomes observed in neonates are due to an overwhelming hyper-inflammatory immune response and/or SII. From the limited data available, it appears that fatal neonatal sepsis may be associated with alterations in immune function that are in agreement with SII findings in adults (3, 4, 6). The dysregulated immune responses observed in various neonatal studies include imbalanced secretion of pro- and anti-inflammatory mediators (53–55), diminished HLA-DR monocyte surface expression (64), expansion of immature neutrophils (70, 71), increased expression of PD-1/PD-L1 (81), and depletion of leukocytes (71, 86, 87). Published neonatal studies are limited by: (i) small sample size of neonates, (ii) incomplete reporting of time from sepsis onset to death, and (iii) the lack of consistent neonatal sepsis definition and objective measures for the degree of severity.

Despite distinct patterns of causative pathogens in adult and neonatal sepsis, similar immune function alterations are observed and endotoxin tolerance appears to be a feature in both adult and neonatal sepsis (9, 35). Therefore, SII may occur independently as a feature of the subsequent host response to inflammation.

In adults, SII is associated with increased susceptibility to secondary bacterial infections and associated late mortality (166, 167). Whether this is a result of organ damage or persistent SII is unclear. Interestingly, one study reported that the immune alterations associated with SII in adult septic shock survivors continued until discharge from the intensive care unit, but resolved by 6 months (168). It is unclear if survivors of neonatal sepsis remain at increased risk of developing subsequent infections (31, 163, 169–171). Preterm infants may have more than one episode of sepsis (~20%) during their NICU admission, and it is uncertain whether having a previous episode of sepsis contributes to the overall risk compared to the major risk factor that is degree of immaturity (31, 163, 169). Preterm neonates, remain at increased risk of infection-related admissions to hospital well into childhood (inversely related to both GA and birth weight) (172), however if neonatal sepsis contributes to infection-related hospital readmissions in childhood has not been studied. Increased risk of subsequent, more severe infections is a hallmark of SII in adults, but it is unclear if this clinical outcome is observed in neonates with sepsis.

## Conclusions

Sepsis mortality in neonates may be associated with alterations in immune function that are in agreement with SII findings in adults. Whether immune cell dysfunction or impairment underpins immunosuppression in neonatal sepsis requires further investigation and stronger evidence. Large, collaborative longitudinal studies, from birth through to childhood, are essential to evaluate immune changes in neonates with sepsis, including the role for SII. Yet, first a definitive consensus on the definition of neonatal sepsis and severity needs to be established. Until then, sepsis severity could be measured by mortality. Advances in further understanding the immunological mechanisms behind immunosuppression may lead to effective targeted treatment therapies for reversing or modulating SII and improve outcomes. Immunosuppression reversal with PD-1/PD-L1 antibody blockade is currently being trialled in adult cancer patients who share similar immune defects as those with SII (151). Furthermore, pentoxifylline, an immune modulatory drug, is currently being trialled to improve long-term outcomes, primarily neurodevelopment impairment, associated with neonatal sepsis (ANZCTR ACTRN12616000405415). Assessing the impact of such interventions on SII-associated markers may provide a mechanistic insight into the success or failure of these interventions in preventing short and long-term negative sepsis outcomes.

## Author contributions

JH conceived and wrote the first and subsequent drafts. TS and AC conceived and revised the manuscript.

### Conflict of interest statement

The authors declare that the research was conducted in the absence of any commercial or financial relationships that could be construed as a potential conflict of interest.
