# Supplementary material for: Sepsis-Induced Immunosuppression in Neonates
Source: Front Pediatr. 2018 Nov 29;6:357. doi: 10.3389/fped.2018.00357 (PMC6281766; doi:10.3389/fped.2018.00357)
Supplement: Supplementary file 1 [file Table_1.docx]

| **SUPPLEMENTARY TABLE 1.** | | **Inflammatory cytokine levels in septic and non-septic neonates without reference to sepsis severity** | | | |
| --- | --- | --- | --- | --- | --- |
| **GA of neonates** | **Cohort sepsis characteristics: n** | | **Time of blood sampling and age at sepsis** | **Observation in septic cohort** | **Reference** |
| Mix of preterm and  Term  (mean GA not described) | Confirmed sepsis: 50   - EOS: 41 - LOS: 9   Non-sepsis inflammation: 50  Controls: 50 | | Blood samples taken at sepsis evaluation (time 0) and on days 1 and 2.  Age at sepsis not described. | IL-6 and IL-8 were significantly higher, at all timepoints, in the sepsis group compared to the other groups.  TNFα was only significantly higher during the first 24 hours. | 54 |
| Mix of preterm and  Term  (mean GA 35.8±4.1) | Confirmed sepsis: 26   - EOS: 13 - LOS: 13   Controls: 29 | | Blood samples taken at sepsis evaluation before antimicrobial therapy (time 0) and on days 3 and 7.  Mean (±SS) age at sepsis:  EOS 1.9 (±1.1) days  LOS 20.6 (±8.4) days | IL-6, IL-8 and TNFα were significantly higher in septic neonates than the controls.  There was no significant difference in IL-8 and TNFα between EOS and LOS. IL-6 was significantly lower in EOS than LOS. | 55 |
| Term  (GA range 37-42 weeks) | Clinical (n=10) and confirmed (n=3) LOS: 13   - Sepsis: 4 - Severe sepsis: 6 - Septic shock: 3 | | Blood sample was taken at initial suspicion of sepsis.  Median age at sepsis: 10 days (IQR 7-22 days) | IL-6, IL-8, IL-10 and IL-1β serum concentrations were higher in the septic infants. TNFα was not elevated. | 53 |
| VLBW  <1500g  (median GA approximately 29 weeks) | Confirmed LOS and NEC: 37   - LOS: 29 - NEC: 8   Non-infected: 90  Controls: 20 | | Blood samples taken at sepsis evaluation (time 0) and at 24 and 48 hours after evaluation.  Age at sepsis not described | IL-2, IL-6, IFNγ, IL-4 and IL-10 were significantly elevated in infected patients at all timepoints.  TNFα was significantly elevated at time 0 and 24 hours only.  IL-6 consistently correlated with TNFα and IL-10. | 94 |
| Preterm and term (mean GA not described) | Confirmed EOS and LOS: 420  (specific numbers of EOS and LOS were not provided)  Controls: 140 | | Blood samples taken at sepsis evaluation (time 0) and following treatment.  Age at sepsis not described | At sepsis onset IL-6, IL-10 and IL-6/IL-10 were significantly elevated in the septic patients.  At sepsis IL-2, IL-4 and TNFα were not elevated.  IL-10 was significantly higher in LOS than EOS, where IFNγ was higher in EOS. No difference with other cytokines.  There was no difference in cytokine levels between Gram-positive and Gram-Negative infections.  Following treatment IL-6 and IL-6/IL-10 significantly decreased. | 95 |
| Preterm and term (mean GA approximately 38 weeks) | EOS: 66   - Confirmed: 13 - Clinical: 36 - Clinical, mother received antepartum antibiotics: 17   Non-sepsis controls: 51 | | Blood sample taken at sepsis evaluation.  Age at sepsis < 5 days after birth | IL-6 and TNFα were significantly higher in the sepsis group. IL-1β levels were not different. | 96 |
| Preterm and term (mean GA not described) | EOS: 10  (preterm n=5 and term n=5)   - Confirmed: 2 - Clinical sepsis: 8   LOS: 15  (preterm n=10 and term n=5)   - Confirmed: 10 - Clinical: sepsis 5   Controls: 8 | | Blood samples taken within 48 hours of clinical onset and 7 days later.  Age at sepsis < 5 days after birth | IL-6 and TNFα were significantly higher in both sepsis groups.  IL-10 was significantly elevated only in LOS.  IL-1β was not elevated in either group.  IL-4 was significantly higher in LOS and significantly lower in EOS than in controls. | 28 |
| Mix of preterm and term (mean GA 32 weeks) | EOS & LOS mix: 56 (preterm n=46 and term n=10)   - Clinical sepsis: 11 - Confirmed: 45   Controls: 41 | | Blood sample taken at initial suspicion of sepsis. Due to experimental treatment, results from other timepoints are not included.  Mean age at sepsis:  Approximately 9 days | IL-6 was significantly higher in the sepsis group. | 93 |
| Moderate preterm (mean GA 34.6 weeks) and  Very preterm (mean GA 28.7 weeks) | Moderate preterm: 12  (Confirmed n=8)   - EOS: 6 - LOS: 6   Very preterm: 14  (Confirmed n=8)   - EOS: 6 - LOS: 8 | | Blood samples were taken 1 week before onset, within 2 days of onset and 1 week after sepsis onset.  Age at sepsis < 30 days after birth | At time of sepsis, IL-6, TNFα and INFγ were elevated in the moderate preterm neonates only.  IL-10 and IL-4 were elevated in both groups at the time of sepsis. | 92 |

GA – gestational age; LOS – late-onset sepsis; EOS – early-onset sepsis; VLBW – very low birth weight; IL – interleukin; TNFα – tumour necrosis factor alpha; IFNγ – type II interferon; NEC – necrotising enterocolitis.

| **SUPPLEMENTARY TABLE 2.** | | **Monocyte surface HLA-DR expression in septic and non-septic neonates without reference to sepsis severity** | | | |
| --- | --- | --- | --- | --- | --- |
| **GA of neonates** | **Cohort sepsis characteristics: n** | | **Time of blood sampling and age at sepsis** | **Observation in septic cohort** | **Reference** |
| Mix of preterm and  Term  (mean GA 37.5±3.8 weeks) | LOS: 40   - Clinical: 22 - Confirmed: 18   Non-sepsis disorder: 24  Controls: 25 | | Blood sample was taken at initial suspicion of sepsis.  Mean age at sepsis: 16 days | Significantly lower HLA-DR expression in septic neonates.  No significant difference between term and preterm.  No significant difference between clinical and confirmed LOS. | 64 |
| Mix of moderate preterm and term (median GA 36; IQR 32-39 weeks) | EOS and LOS: 63   - Clinical: 42 - Confirmed: 21   Non-sepsis: 37  Controls: 29 | | Blood sample was taken at initial suspicion of sepsis.  Median age at sepsis: 4 days | HLA-DR expression was significantly decreased in septic neonates.  Differences between EOS and LOS, and clinical and confirmed sepsis were not described. | 65 |
| Mix of preterm and term (mean GA 32 weeks) | EOS & LOS: 56  (preterm n=46 and term n=10)   - Clinical: 11 - Confirmed: 45   Controls: 41 | | Blood sample was taken at initial suspicion of sepsis. Due to experimental treatment, results from other timepoints are not included.  Mean age at sepsis: approximately 9 days | Significantly lower HLA-DR expression in septic neonates.  No significant difference between term and preterm.  Differences between clinical and confirmed sepsis were not described. | 93 |
| Preterm (mean GA 31±2 weeks)  and  term (mean GA 39±1 weeks) | LOS: 44  (preterm n=33 and term n=11)   - Clinical: 7 - Confirmed: 37   Non-sepsis RDS: 20  Non-sepsis perinatal asphyxia: 8  Controls: 59  (36 preterm & 23 term)  Adults: 20 | | Blood sample was taken at initial suspicion of sepsis.  Mean (±SE) age at sepsis:  Preterm 9±7 days  Term 6±2 days | Significantly lower HLA-DR expression in septic neonates.  No significant difference between term and preterm.  Differences between clinical and confirmed sepsis were not described. | 112 |
| Preterm (mean GA 31±2 weeks) | EOS: 22  Controls: Not described | | Blood samples taken at admission to NICU during first 48 hours of life, during infection, and recovery.  Mean age at sepsis: Not described. | HLA-DR expression significantly decreased in neonates with perinatal infection compared to neonates without infection. | 66* |

HLA-DR – Human Leukocyte Antigen-DR isotype; EOS- early-onset sepsis; LOS – late-onset sepsis; NICU – Neonatal Intensive Care Unit; RDS – respiratory distress syndrome; NICU – neonatal intensive care unit; *- conference abstract only, limited data available.
